# Accelerated Super-Resolution Reconstruction for Structured Illumination Microscopy Integrated with Low-Light Optimization

**DOI:** 10.3390/mi16091020

**Published:** 2025-09-03

**Authors:** Caihong Huang, Dingrong Yi, Lichun Zhou

**Affiliations:** 1 College of Information Science and Engineering, Huaqiao University, Xiamen 361021, China; 2 College of Mechanical Engineering and Automation, Huaqiao University, Xiamen 361021, China; yidr@hqu.edu.cn (D.Y.); 13905958730@163.com (L.Z.)

**Keywords:** structured illumination microscopy, signal processing, super-resolution reconstruction, low-light image enhancement

## Abstract

Structured illumination microscopy (SIM) with π/2 phase-shift modulation traditionally relies on frequency-domain computation, which greatly limits processing efficiency. In addition, the illumination regime inherent in structured illumination techniques often results in poor visual quality of reconstructed images. To address these dual challenges, this study introduces DM-SIM-LLIE (Differential Low-Light Image Enhancement SIM), a novel framework that integrates two synergistic innovations. First, the study pioneers a spatial-domain computational paradigm for π/2 phase-shift SIM reconstruction. Through system differentiation, mathematical derivation, and algorithm simplification, an optimized spatial-domain model is established. Second, an adaptive local overexposure correction strategy is developed, combined with a zero-shot learning deep learning algorithm, RUAS, to enhance the image quality of structured light reconstructed images. Experimental validation using specimens such as fluorescent microspheres and bovine pulmonary artery endothelial cells demonstrates the advantages of this approach: compared with traditional frequency-domain methods, the reconstruction speed is accelerated by five times while maintaining equivalent lateral resolution and excellent axial resolution. The image quality of the low-light enhancement algorithm after local overexposure correction is superior to existing methods. These advances significantly increase the application potential of SIM technology in time-sensitive biomedical imaging scenarios that require high spatiotemporal resolution.

## 1. Introduction

Microscopy is indispensable in life sciences, enabling landmark biological discoveries. Nonetheless, the diffraction barrier, initially outlined by Ernst Abbe in 1873, has historically confined optical resolution to approximately half the wavelength of the employed light [1]. The emergence of super-resolution microscopy (SRM) techniques has overcome this limitation, among which structured illumination microscopy (SIM) achieves twofold resolution enhancement via patterned excitation and computational reconstruction [2,3].

SIM offers distinct advantages, including rapid imaging, minimal phototoxicity, and compatibility with standard sample preparation, making it a powerful tool for biomedical research [4,5,6,7].

Despite its strengths, SIM reconstruction relies heavily on Fourier-domain processing, which imposes computational bottlenecks that hinder real-time imaging [8]. Conventional frequency-domain reconstruction (FDR) requires iterative parameter optimization (e.g., precise frequency/phase estimation), further delaying processing [9,10,11]. While recent advances accelerate reconstruction via hardware innovations [12], algorithmic refinements [13], or deep learning [14,15,16,17,18,19,20], spatial-domain methods have demonstrated exceptional efficiency gains. SP-SIM and functional expansion approaches achieve > 5× faster processing than FDR, yet both require a 2π/3 phase shift [21]. Critically, this restricts their application in high-resolution DMD-based SIM systems where illumination geometry mandates a π/2 phase shift to maximize spatial frequency near the diffraction limit [22]. Notably, π/2 shifting improves reconstruction accuracy under low modulation conditions, highlighting its importance for high-fidelity imaging [22]. In this study, the spatial domain reconstruction (SDR) method of structured lighting with π/2 phase difference will be studied.

Furthermore, SIM’s low-light illumination inherently compromises image contrast and visual quality. While low-light enhancement (LLIE) algorithms based on Retinex theory or deep learning exist [23], their applicability to SIM remains underexplored. The RUAS network [24] holds promise for enhancing SIM but is plagued by non-uniform illumination artifacts. These artifacts lead to overenhancement in bright regions and insufficient recovery of dark regions [25].

This limitation is particularly problematic in fluorescence microscopy, where sample sparsity and optical heterogeneity inherently produce intensity variations.

To address these dual challenges, the study proposes DM-SIM-LLIE, an integrated computational framework featuring the following:

(1) A novel spatial-domain reconstruction (SDR) algorithm optimized for π/2 phase-shifted SIM, eliminating FDR bottlenecks while enhancing axial resolution;

(2) An adapted RUAS network with dynamic estimate truncation, smoothness constraints, and attention mechanisms, specifically engineered to correct non-uniform illumination in SIM reconstructions.

## 2. Methods and System

### 2.1. Principle of DM-SIM

In the linear SIM system, cosine fringe structured illumination is used. Let *S*(*r*) represent fluorophore density distribution within a specimen and $I_{\theta ,\varphi }(r)$ be the illuminating sinusoidal intensity pattern,

(1) $$I_{\theta ,\varphi }(r)=I_{0}[1+m\cdot \cos(2\pi k_{0}\cdot r+\varphi )]$$

where r≡(x,y) is the (two-dimensional) spatial position vector, *k*_0_ is the (sinusoidal) illumination frequency vector in reciprocal space, and *φ* denotes the phase of the sinusoidal fringe illumination pattern [11]. *I*_0_ and m constants are called the mean intensity and modulation depth, respectively [26].

Thus, the fluorescence emission distribution from the specimen is $S(r)\cdot I_{\theta ,\varphi }(r)$, and the observed emission distribution through the optical system is as follows:

(2) $$D_{\theta ,\varphi }(r)=[I_{\theta ,\varphi }(r)\cdot S(r)]⊗H(r)+B_{\mathrm{out}}$$

where *H*(*r*) *is the optical system*’s point spread function (PSF), ⊗ is the convolution operator, and *Bout* accounts for out-of-focus background contributions.

If making Fourier transform, Equation (2) can be expressed in frequency space as Equation (3) [27].

(3) $$\begin{array}{cc} {\tilde{D}}_{\theta ,\varphi }(k) & =[{\tilde{I}}_{\theta ,\varphi }(k)⊗\tilde{S}(k)].\tilde{H}(k)+{\tilde{B}}_{out}(k)\\ & =I_{0}[{\tilde{S}}_{w}(k)+\frac{m}{2}\tilde{S}(k+k_{0})e^{-i\varphi }+\frac{m}{2}\tilde{S}(k-k_{0})e^{i\varphi }]\cdot \tilde{H}(k) \end{array}$$

where ${\tilde{S}}_{w}(k)=\frac{\tilde{S}(k)\tilde{H}(k)+{\tilde{B}}_{\mathrm{out}}(k)}{\tilde{H}(k)}$, $\tilde{H}(k)$ is the system Optical Transfer Function (OTF). ${\tilde{S}}_{w}(k)$ in Equation (3) characterizes the standard frequency spectrum detectable through conventional microscopy, where the spatial frequency k is constrained within the range of [−*kc*, *kc*], with kc denoting the system’s optical cutoff frequency. Notably, Equation (3) incorporates two supplementary spectral components. These additional components exhibit distinct spectral shifts. One is displaced by +*k*_0_ and the other by −*k*_0_ along the frequency axis, while their respective k values satisfy modified boundary conditions. This spatial frequency redistribution effectively expands the observable spectral range to [−*kc* − *k*_0_, *kc* + *k*_0_]. Given that *k*_0_ can theoretically approach kc, the maximum achievable spectral bandwidth in linear SIM configurations demonstrates a two-fold expansion compared to conventional systems. This fundamental principle forms the basis for the twofold resolution enhancement characteristics of linear SIM techniques. To obtain the three terms of frequency spectra in Equation (3), three different phases, *φ*_0_, *φ*_0_ + Δ*φ*, and *φ*_0_ + 2Δ*φ*, for the illumination pattern can be set to get three independent linear equations. To obtain nearly isotropic resolution enhancement, the unidirectional sinusoidal fringe pattern should be rotated in at least two orthogonal directions or in three directions, such as *θ*, *θ* + *π*/3, and *θ* + 2*π*/3.

**Figure 1:**
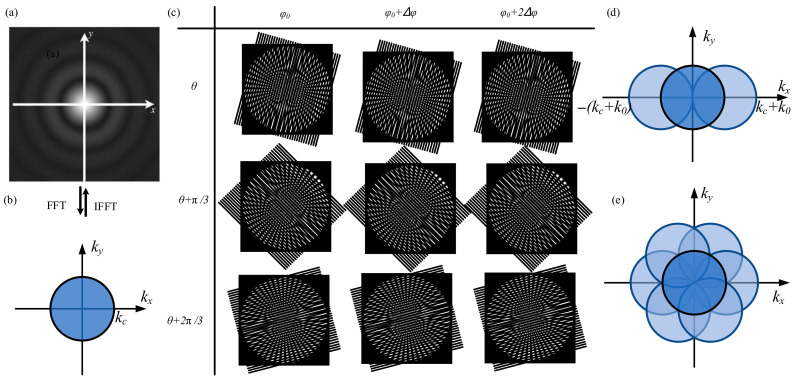
Schematic diagram of SIM foundation.

The schematic diagram of the SIM foundation is shown in Figure 1 [27].

Figure 1a The PSF of a microscope, which can be obtained from system calibration. The OTF and the PSF are a Fourier transform pair. FFT, Fourier transform; IFT, inverse Fourier transform. Figure 1b The OTF of a conventional wide-field microscope, which shows the observable region in the frequency domain. Moiré patterns reveal detailed structural information. When two fringes superpose on each other, low-frequency fringes will appear. Different angles of superposition lead to moiré fringes of different directions and frequencies. As shown in different rows of Figure 1c, the fringe angle is rotated by π/3 to generate fringes in three directions. These fringes extend the cutoff frequency of the OTF in the corresponding directions. In each direction, fringes are phase shifted three times with a step of Δ*φ* figures in the same row in Figure 1c. Figure 1d A horizontal sinusoidal pattern with a spatial frequency kc expands the observable region Figure 1e. Achieving isotropic resolution enhancement by rotating the structured illumination pattern.

The study employs the relative spatial phases *φ*_1,1_ = *φ*_0_, *φ*_1,2_ = *φ*_0_ + Δ*φ* = *π*/2, and *φ*_1,3_ = *φ*_0_ + 2Δ*φ* = *π*. Using Equation (1) and combining the properties of trigonometric functions, we can obtain the expression of the three captured images.

(4) $$\begin{array}{c} D_{1,1}(r)=\left\{I_{0}[1+m\cdot \cos(2\pi k_{0}\cdot r+\varphi_{0})]\cdot S(r)\right\}⊗H(r)+B_{\mathrm{out}}\\ D_{1,2}(r)=\left\{I_{0}[1-m\cdot \sin(2\pi k_{0}\cdot r+\varphi_{0})]\cdot S(r)\right\}⊗H(r)+B_{\mathrm{out}}\\ D_{1,3}(r)=\left\{I_{0}[1-m\cdot \cos(2\pi k_{0}\cdot r+\varphi_{0})]\cdot S(r)\right\}⊗H(r)+B_{\mathrm{out}} \end{array}$$

Since the out-of-focus background remains unchanged, the background can be eliminated by subtracting two neighboring phase-shifted images. Further simplification is carried out using the formula for the sum and difference of two angles of a trigonometric function, and the result is shown in Equation (5).

(5) $$\begin{array}{ccc} \Delta D_{1,1,1,2}(r) & = & D_{1,1}(r)-D_{1,2}(r)\\ & = & =\sqrt{2}m\cdot I_{0}\cdot \{[\cos(2\pi k_{0}\cdot r+\varphi_{0})\cos\frac{\pi }{4}+\\ & & \sin(2\pi k_{0}\cdot r+\varphi_{0})\sin\frac{\pi }{4}]\cdot S(r)\}⊗H(r)\\ & = & \sqrt{2}m\cdot I_{0}[\cos(2\pi k_{0}\cdot r+\varphi_{0}-\frac{\pi }{4})\cdot S(r)]⊗H(r)\\ \Delta D_{1,2,1,3}(r) & = & D_{1,2}(r)-D_{1,3}(r)\\ & = & \sqrt{2}m\cdot I_{0}\cdot \{[\sin(2\pi k_{0}\cdot r+\varphi_{0})\cos\frac{\pi }{4}-\\ & & \cos(2\pi k_{0}\cdot r+\varphi_{0})\sin\frac{\pi }{4}]\cdot S(r)\}⊗H(r)\\ & = & -\sqrt{2}m\cdot I_{0}[\sin(2\pi k_{0}\cdot r+\varphi_{0}-\frac{\pi }{4})\cdot S(r)]⊗H(r) \end{array}$$

Euler’s formula, $e^{i\cdot r}=\cos(r)+i\cdot \sin(r)$, suggests the construction of a complex analytic signal, with $\Delta D_{1,1,1,2}(r)$ as the real part and $\Delta D_{1,2,1,3}(r)$ as the imaginary part. The following simplification via Euler’s formula directly yields Equation (6).

(6) $$\begin{array}{ccc} Z_{1}(r) & = & \Delta D_{1,1,1,2}(r)-i\Delta D_{1,1,2,3}(r)\\ & = & \sqrt{2}m\cdot I_{0}[\cos(2\pi k_{0}\cdot r+\varphi_{0}-\frac{\pi }{4})\cdot \sin(r)]⊗H(r)\\ & + & i\sqrt{2}m\cdot I_{0}[\sin(2\pi k_{0}\cdot r+\varphi_{0}-\frac{\pi }{4})\cdot \sin(r)]⊗H(r)\\ & = & \sqrt{2}[m\cdot I_{0}e^{i\cdot (2\pi k_{0}r+\varphi_{0}-\pi /4)}\cdot S(r)]⊗H(r) \end{array}$$

A local analysis of Equation (6) is performed according to the convolution formula [19], letting $t=r-t^{\prime }$.

(7) $$\begin{array}{cc} \left[e^{i2\pi k_{0}r}\cdot S(r)]⊗H(r\right) & =\int_{-\infty }^{\infty }e^{i\cdot 2\pi k_{0}(t)}\cdot S(t)\cdot H(r-t)dt\\ & =\int_{-\infty }^{\infty }e^{i\cdot 2\pi k_{0}(r-t^{\prime })}\cdot S(r-t^{\prime })\cdot H(t^{\prime })dt^{\prime }\\ & =e^{i\cdot 2\pi k_{0}r}\int_{-\infty }^{\infty }S(r-t^{\prime })\cdot H(t^{\prime })e^{-i\cdot 2\pi k_{0}r}dt^{\prime }\\ & =e^{i\cdot 2\pi k_{0}r}\cdot [S(r)⊗(H(r)e^{-i\cdot 2\pi k_{0}r})] \end{array}$$

According to Equation (7), combined with the Fourier transform frequency shift property, Equation (6) can be simplified to Equation (8).

(8) $$Z_{1}(r)=\sqrt{2}I_{0}e^{i\cdot (2\pi k_{0}r+\varphi_{0}-\pi /4)}[S(r)⊗(H(r)\cdot e^{-i\cdot 2\pi k_{0}r})]$$

It is known from Euler’s formula and the properties of complex numbers that $\left|e^{i\theta }\right|=1$. Then, a super-resolution image of the sample with spatial frequency $\left|k|\epsilon (0,|k_{0}|+k_{c}\right)$ would be obtained by taking the modulus of *Z*_1_ (*r*), as shown in Equation (9).

(9) $$\begin{array}{cc} \left|Z_{1}(r)\right| & =\left|\sqrt{2}mI_{0}e^{i\cdot (2\pi k_{0}r+\varphi_{0}-\pi /4)}\left[S(r)⊗\left(H(r)e^{-i\cdot 2\pi k_{0}r}\right)\right]\right|\\ & =\sqrt{2}mI_{0}\left|S(r)⊗\left(H(r)\cdot e^{-i\cdot 2\pi k_{0}r}\right)\right|\\ & =\sqrt{2}mI_{0}\left|FT^{-1}[\tilde{s}(k)\tilde{h}(k-k_{0)}]\right| \end{array}$$

The above excitation mode angles are only for solving the super-resolution problem at one *θ* angle. To optimize the uniformity of spatial resolution, the reconstructed volumetric data should be enhanced across all orientations. The system performs fluorescence wide-field imaging with different phase values in three directions (i.e., different cosine modulation k0 values). The evolution images $Z_{i}(r)$ (*i* = 1, 2, 3) are obtained in sequence. Finally, it can obtain a 2D HR image *D*(*r*) by summing up the $Z_{i}(r)$ (*i* = 1, 2, 3) in three structured lighting *θ* angles of 0°, 60°, and 120°.

(10) $$D(r)=\sum_{i=1}^{3}\left|Z_{i}(r)\right|$$

### 2.2. LLIE Principle and Improved Illumination Estimation Method

Based on the fundamental principles of Retinex theory, the RUAS computational framework performs image decomposition through dual-component separation. In the process of illumination estimation, the algorithm mainly relies on a local maximum to estimate the initial illumination component, although the algorithm uses a convolutional neural network to further optimize the illumination component to adapt to the situation of non-uniform illumination. However, this method still has the following shortcomings:

- Local overexposure problem: Under non-uniform illumination conditions, the local maximum may correspond to the pixel value of the overexposed area. Directly using these values as the initial illumination estimation will cause the overexposed area to be over-magnified during the enhancement process, resulting in local overexposure.
- Insufficient illumination smoothness: The spatial change of illumination components should have a certain smoothness, but the local maximum estimation method of the RUAS algorithm cannot effectively constrain the smooth transition of illumination components, and it is easy to produce mutations in local areas, further exacerbating the overexposure problem.

This study proposes an improved illumination estimation method that combines global constraints, dynamic range adjustments, and adaptive optimization to suppress the enhancement distortion of overexposed areas.

Step 1: Dynamically truncate the initial illumination estimate.

Goal: Avoid using the pixel values of overexposed areas as initial illumination estimates.

First, detect the overexposed areas. Scale the intensity values of each image channel to the normalized range between 0 and 1.

The input image will be segmented into n × n non-overlapping matrix blocks. In this study, four non-overlapping block sizes (i.e., 2 × 2, 4 × 4, 6 × 6, and 8 × 8) were used to segment the image. The purpose of dividing the blocks into different sizes is to analyze the exposure of the image from different scales.

Next, a parameter *f_bc_* called the block exposure fitness is introduced.

(11) $$f_{bci}=\frac{\sqrt{n\times n\sum_{j=1}^{n\times n}{\left(V_{i,j}-{\bar{V}}_{i}\right)}^{2}}}{n\times n}$$

The luminance value at position (*i*,*j*) within a given image block is denoted as *V*_*i*,*j*_, while $\bar{V_{i}}$ represents the mean intensity across the n × n pixel area of the corresponding block. The feature *f_bci_* is computed through a contrast-based calculation that evaluates the deviation of individual pixel intensities from their local mean. This metric is normalized to the interval [0, 1], with values approximating 0.5 indicating optimal visual quality and natural appearance. Regions exhibiting *f_bci_* values exceeding 0.65 are identified as potential overexposure areas requiring correction.

(12) $$Exposure=\left\{\begin{array}{cc} \text{Well-expose} & \mathrm{if}\;0\leq f_{bci}\leq 0.65\\ \text{Over-expose} & \mathrm{if}\;0.66\;\leq f_{bci}\leq 1.0 \end{array}\right.$$

Assign weights to the results of different segmentation block sizes. Smaller blocks get higher coefficients to amplify local brightness details, and larger blocks get less weights for overall brightness evaluation. Then, an illumination matrix is generated to determine whether each pixel of the image is overexposed.

Step 2: Lighting smoothness constraint.

Goal: Suppress the sudden change of illumination components and enhance the robustness to non-uniform illumination.

(13) $$L_{TV}=\sum_{x_{i},y_{j}}\left(\left|\frac{\partial L}{\partial_{x_{i}}}\right|+\left|\frac{\partial L}{\partial_{y_{j}}}\right|\right)$$

Add a total variation (*TV*) regularization term to the loss function. Where *L* represents the illumination component, and *x_i_* and *y_j_* represent the horizontal and vertical coordinates of the image, respectively. When optimized by a convolutional neural network, the gradient of the illumination component is forced to be smooth.

Step 3: Adaptive attention mechanism.

Goal: Reduce the weight of overexposed areas in the illumination estimation. Make the enhancement algorithm pay more attention to normal areas.

According to the illumination matrix, an attention mechanism map A is generated, where the weight of overexposed areas is low (A = 0.1) and the weight of normal areas is high (A = 1.0). During the optimization process, give less weight to the loss of overexposed areas.

**Figure 2:**
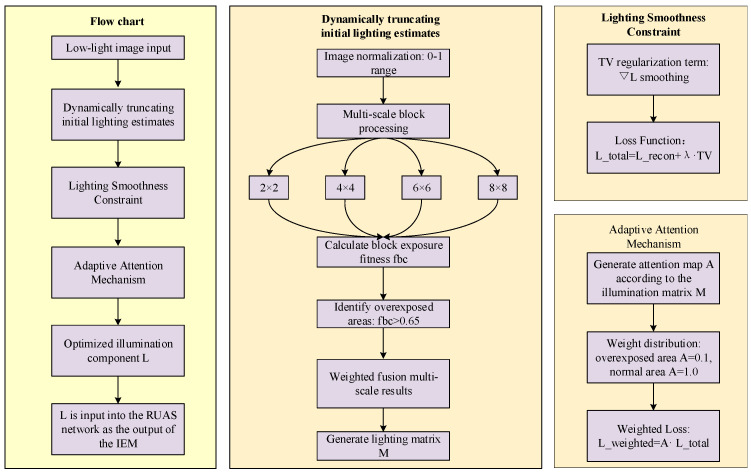
Network structure diagram of the improved lighting estimation method.

The optimized illumination component L will be used as the output of the IEM (Illumination Estimation Module) of the improved RUAS network. The network structure diagram of the improved lighting estimation method is shown in Figure 2.

### 2.3. Flowchart of DM-SIM-LLIE Method

**Figure 3:**
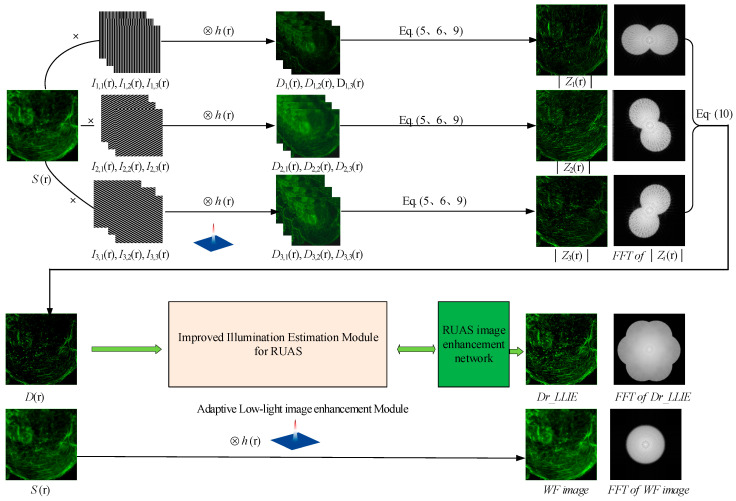
Flowchart of DM-SIM-LLIE.

A flowchart of our DM-SIM-LLIE is presented in Figure 3. The sample *S*(*r*) is sequentially illuminated by cosine fringes with different directions and phase shifts. A total of nine original images (three directions and three phase shifts for each direction) are recorded. *D*_*i*,*j*_(*r*) represents the captured image associated with the illumination pattern of the i-th orientation and j-th phase displacement, where *i*, *j* = 1, 2, 3. The DM-SIM algorithm sequentially processes each orientation to generate corresponding super-resolved sub-images. Finally, the overall two-dimensional super-resolution image *D*(*r*) is obtained by summing the three reconstructed sub-images across the three directions. The spectrograms of the images $\left|Z_{i}(r)\right|$ and *D*(*r*), along with the wide-field image of the microscope are shown simultaneously in Figure 3. This visualization effectively demonstrates the spectral expansion mechanism in the spatial frequency domain. Given that the parameter k0 reaches its theoretical maximum at the system’s cutoff frequency kc, the reconstruction process enables a doubling of the lateral resolution relative to standard microscopy configurations through an optimized manipulation of the optical transfer function. This finding is consistent with the theoretical resolution enhancement results of the frequency domain method of SIM.

The super-resolution image *D*(*r*) reconstructed by DM-SIM is input into the illumination estimation module of RUAS optimized by this study, and an attention mechanism map is generated. This encourages the enhancement algorithm to pay more attention to the non-overexposed areas, i.e., the normal areas, and to perform more effective low-light enhancement.

## 3. Experimental System Setup

**Figure 4:**
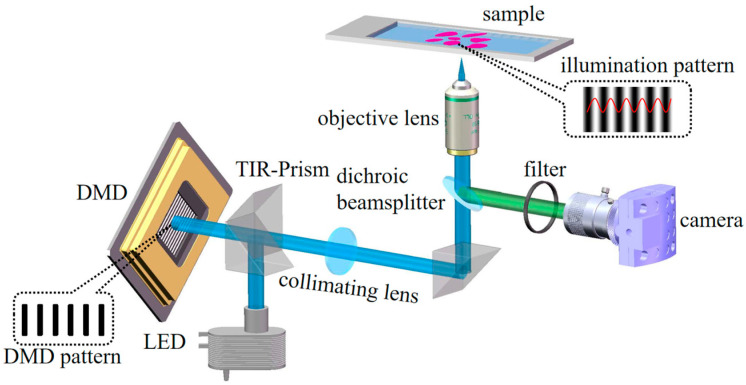
Schematic of the SIM system.

The experiment was carried out by employing a SIM system that relied on LED illumination and DMD projection [28]. Figure 4 illustrates the experimental setup, featuring a multi-wavelength LED array with bandpass filters for excitation. Light passes through a TIR prism, reflects off a DMD chip programmed with periodic grating patterns, and is projected via a collimating lens and objective to create a cosine grating illumination. Image acquisition is performed using an sCMOS camera. DMD is a high-speed digital switching mirror array consisting of millions of micrometre-square aluminium micromirrors integrated on a memory chip to form a two-dimensional array, with each micromirror representing a pixel. Each pixel can be individually controlled, allowing binary stripes with different phases; angles and periods can be rapidly loaded without mechanical movement or rotation. Because of the low-pass filtering characteristic of the microscope system, only sinusoidal stripes with a fundamental frequency not exceeding the cut-off frequency can smoothly reach the focal plane, while higher-order harmonics are blocked. This process effectively transforms DMD binary stripes into sinusoidal stripes of the same frequency.

## 4. Results and Discussion

The following experiments will target different samples, sequentially obtaining wide-field images and reconstructed images of structured light illumination microscopy to verify the effectiveness of the algorithm. Part of the experimental process can be referenced in Figure 3. Wide-field imaging is a technique that captures images of a large area of a sample at once, rather than focusing on a small section, providing a comprehensive view of the whole. In microscopy, this means illuminating the entire sample and observing the image through the eyepieces or a digital camera. The SIM image acquisition process is carried out as follows: Instead of uniform light, SIM illuminates the sample with a patterned light, often a grid of stripes or a similar structure. When the patterned light interacts with the biological sample, it creates interference patterns called Moiré fringes. These Moiré fringes encode fine-scale information about the sample’s structure that is normally invisible under standard illumination due to the diffraction limit. Multiple images are captured with different patterns (e.g., rotated or phase-shifted light patterns). Software then analyzes these images and the known illumination patterns to computationally reconstruct a high-resolution image, effectively “unmixing” and extracting the encoded high-frequency information.

### 4.1. Improved Lateral Resolution Results and Analysis

A practical way to quantify resolution is the full width at half maximum (FWHM) of sub-diffraction-limited structures. This metric, easily obtainable through microscopy, serves as a standard for comparative analysis. The theoretical FWHM is given by FWHM = 0.51λ/(NA), approximately equal to λ/(2NA). Thus, the FWHM closely approaches Abbe’s diffraction limit and can also be derived from microscope image data.

**Figure 5:**
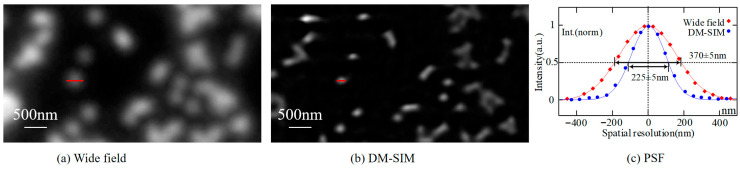
System resolution was calibrated using fluorescent microspheres with a diameter of 200 nm. (**a**) Wide-field reconstruction. (**b**) DM-SIM reconstruction. (**c**) FWHM of wide-field and DM-SIM system.

The spatial resolution calibration protocol employed 200 nm diameter fluorescent beads from TetraSpeck (ThermoFisher). The results of the wide-field and DM-SIM measurements are presented in Figure 5. This work selected individual beads to plot their intensity distributions, as shown in Figure 5a,b, and fitted these distributions with a Gaussian function to obtain FWHM. The average FWHM values for wide-field and DM-SIM measurements are 370 ± 5 nm and 225 ± 5 nm, respectively, as illustrated in Figure 5c. The error components take into account both systematic errors and random errors. The resolution of the DM-SIM reconstruction is approximately 1.64 times that of the wide-field image. Given that the size of a DMD micromirror pixel used in the experimental system is 13.68 μm, and that four DMD pixels are used in one cycle, the size of one cycle after passing through the objective lens is approximately 548 nm. Consequently, the frequency of the structured illumination stripes is about 0.66 times the diffraction limit, with a theoretical resolution expected to be 1.66 times that of the wide-field resolution. Therefore, the experimental results are close to the theoretical values. If a DMD with smaller micromirror sizes is utilized in future experiments, DM-SIM is expected to achieve a system resolution close to twice that of the wide-field resolution, aligning with theoretical predictions.

### 4.2. Low-Light Image Enhancement Verification

BioSR is an open experimental dataset. Researchers utilized a multimodal SIM (Structured Illumination Microscopy) approach to collect a comprehensive dataset encompassing various cellular components, including clathrin-coated pits (CCPs), the endoplasmic reticulum (ER), microtubules (MTs), and F-actin filaments [29,30]. This study used ccps, MTs, and F-actin samples in the data set to verify our LLIE algorithm.

**Figure 6:**
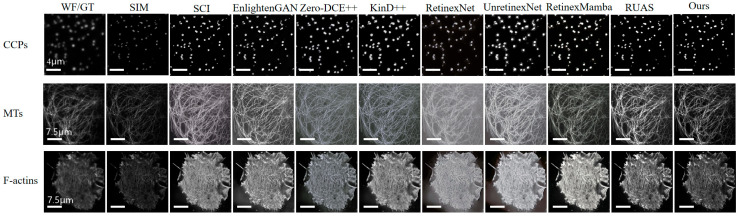
Visual comparison of the Bio dataset among advanced low-light image enhancement approaches.

Figure 6 presents a comprehensive comparison of our approach, with various advanced methods across both supervised and unsupervised learning frameworks. The comparative analysis relies on datasets sourced from the BioSR repository. Usually, image quality is assessed by full reference metrics. Performance Evaluation is shown in Table 1. Structural Similarity Index (SSIM), Root Mean Square Error (RMSE), Contrast Improvement Index (CII), and Peak signal-to-noise ratio (PSNR) are selected as the parameters for image evaluation. SSIM assesses the structural similarity between two images by considering luminance, contrast, and structure. It is often used to evaluate the perceived quality of an image compared to a reference image. To measure the amount of improvement in contrast and brightness, the contrast improvement index (CII) was used. PSNR is a measure of the quality of a compressed or reconstructed image compared to the original image. It quantifies how much noise or distortion is present. The root mean square error is defined as the measure of the differences between values that are predicted by a model and values that are actually observed. Image quality improves with higher SSIM, PSNR, and CII values. In RMSE results for images, lower values are better.

Here, the study mainly selects some low-light enhancement algorithms that are advanced but do not require very high hardware resources for comparison. The comparison algorithms shown here mainly include the following: the self-calibrated illumination (SCI) learning framework introduced by Ma et al. [31]; an unsupervised generative adversarial network for LLIE (Englighten-GAN) [32]; Zero-Reference Deep Curve Estimation for Low-Light Image Enhancement (Zero-DCE++) [33]; the low-light enhancement algorithm KIND++ with a multi-scale illumination attention module that has been introduced [34]; the low-light image enhancement convolutional neural network RetinexNet [35]; URetinex-Net: Retinex-based Deep Unfolding Network for Low-light Image Enhance [36]; and Retinexmamba: Retinex-based Mamba for Low-light Image Enhancement [37]. For the CCPS dataset, the proposed method achieved the highest scores in both SSIM and PSNR metrics. The CII metric was lower than most algorithms but higher than the RetinexNet algorithm. RMSE achieved the lowest score in the comparison, indicating the best results. Experiments on MTs samples yielded similar results, with the CII metric outperforming RetinexNet, ZeroDCE++, and Kind++ algorithms. Experiments on F-actin samples also achieved the best results among all algorithms in terms of SSIM, PSNR, and RMSE parameters. However, the CII metric was lower than that of other algorithms. Overall, the results of this study are satisfactory. This study’s proposed algorithm produces the most robust image enhancement effect.

In particular, some of the image enhancement algorithms produce blurred backgrounds. A few algorithms introduced artifacts.

### 4.3. Image Reconstruction Results After Combining the LLIE Process

**Figure 7:**
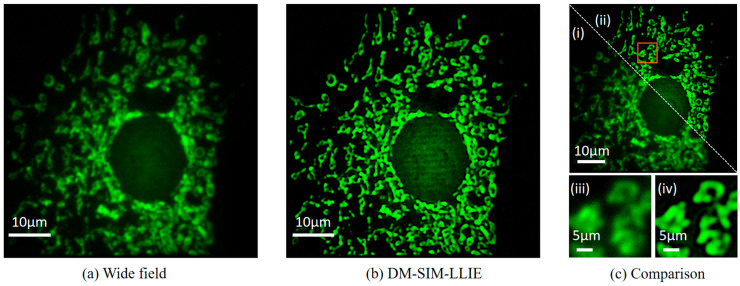
Performance of DM-SIM combining the LLIE process on reconstructed BPAE cell. (**a**) Wide-field; (**b**) DM-SIM-LLIE; (**c**) Comparison between wide-field and the DM-SIM-LLIE method.

Next, the study compared the imaging results under the traditional illumination mode (i.e., wide-field illumination mode) with the imaging results of the DM-SIM algorithm combining the LLIE process. As shown in Figure 7, (a) is the image of BPAE cells under wide-field conditions, and (b) is the image of the DM-SIM algorithm combining the LLIE process. (c) compares (a) and (b) together. (i) is the wide-field-of-view image, and (ii) is the DM-SIM image. (iii) and (iv) are magnified images with red rectangular boxes. In contrast, DM-SIM incorporates the LLIE process to automatically acquire clear super-resolution reconstructed images. It overcomes the shortcomings of the traditional SIM method, which is affected by low illumination and requires manual contrast adjustment in the reconstructed image.

### 4.4. Comparison of DM-SIM-LLIE with FDR-SIM

In the traditional FDR-SIM scheme, super-resolution images are achieved through Fourier transform and spatial spectrum processing. FairSIM is a commonly used program for reconstructing super-resolution images, and its principle is based on the frequency domain method [38]. The study used the FairSIM program to perform structured light illumination super-resolution reconstruction based on the frequency domain method, which is the FDR-SIM scheme.

**Figure 8:**
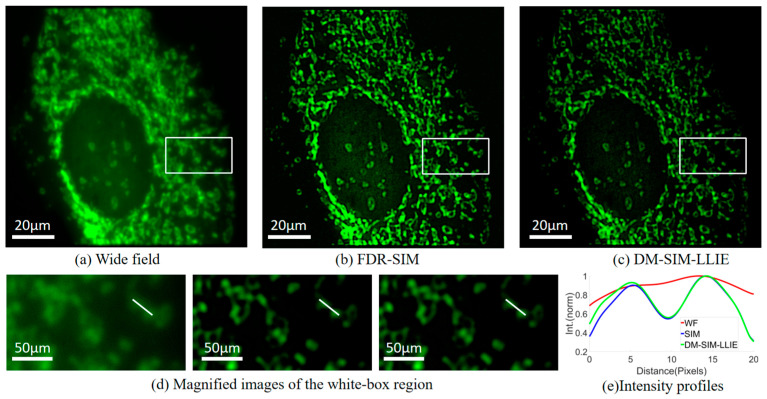
Imaging results of BPAE cell. (**a**) Wide-field image. (**b**) FDR-SIM reconstructed image. (**c**) DM-SIM reconstructed image. (**d**) Zoomed-in views of the boxed parts in (**a**–**c**). (**e**) Intensity curves along the marked lines in (**d**).

To determine the comparison between DM-SIM and FDR-SIM, the study still selected BPAE cells (F36924, ThermoFisher) as samples. The reconstructed image shown in Figure 8a is the wide-field image, Figure 8b is reconstructed by FairSIM, and Figure 8c is reconstructed by DM-SIM. Figure 8d is the enlarged image of the white box part in Figure 8a–c, and Figure 8e is the intensity distribution fitting curve along the white line in Figure 8d. Comparison of the enlarged area of each image in Figure 8 shows that the image generated by DM-SIM is very similar to that generated by FairSIM. The contour plot also shows that DM-SIM can recover the same details as FairSIM using the frequency domain method.

Under the same hardware conditions, the image reconstruction time of each reconstruction method is compared, and the advantage of DM-SIM-LLIE is revealed.

Details are given in Table 2. The reconstruction time of DM-SIM for a 512 × 512 pixel image was 0.71 s. In comparison, FDR-SIM’s reconstruction time was 5.49 times slower at 3.90 s. In the aspect of improving low illumination, the traditional way is manual adjustment, which is easily influenced by human factors. DM-SIM-LLIE, on the other hand, achieves ideal time efficiency.

### 4.5. Improved Axial Resolution Results and Analysis

Wide-field fluorescence microscopy suffers from poor axial resolution and OTF missing cone issues, causing 2D images to mix in-focus details with out-of-focus blur. Removing this interference is crucial for clear 3D reconstruction.

The following experiments verify that the DM-SIM algorithm has the ability to reduce the interference of out-of-focus signals and improve the axial resolution.

The specimen selection is mouse kidney slices; light source: central wavelength 470 nm; dye: 488 nm wheat germ agglutinin; objective lens: 40×/NA 0.6.

Optical sectioning SIM (OS-SIM) microscopy employs orthogonal light paths to confine illumination to a single plane, enabling inherent optical sectioning. This computational imaging technique is widely regarded as an effective method for enhancing axial resolution.

**Figure 9:**
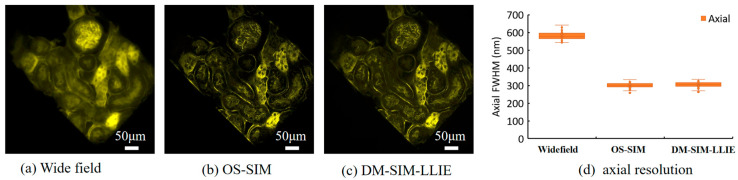
Imaging results of mouse kidney slices. (**a**–**c**) Images by wide-field, OS-SIM, and DM-SIM-LLIE, respectively. (**d**) The axial resolution of WF, OS-SIM, and DM-SIM-LLIE.

Taking mouse kidney sections as specimens, Figure 9a–c compare the imaging results of the specimens under wide-field microscopy, OS-SIM, and DM-SIM. The results show that, similar to OS-SIM, DM-SIM can suppress defocused background noise, ensuring clarity in focal plane information.

Compared with wide-field imaging, the focal plane information resolution of DM-SIM is improved. Figure 9d is a comparison of the axial resolution of WF, OS-SIM, and DM-SIM. The axial resolution of WF is 580 ± 5 nm, the axial resolution of OS-SIM is 300 ± 5 nm, and the axial resolution of DM-SIM is 305 ± 5 nm.

DM-SIM has achieved an axial resolution close to that of OS-SIM, which is an advantage that traditional frequency domain methods do not have. This is mainly because the image differential process has the effect of eliminating defocus interference.

### 4.6. Experimental Evaluation on Different Samples

**Figure 10:**
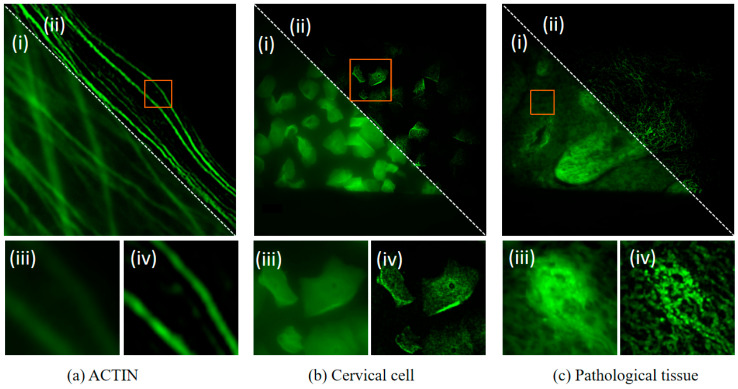
Experimental evaluation on different samples. (**a**) ACTIN sample. (**b**) Cervical cell sample. (**c**) Pathological tissue sample.

In addition to the above experiments on animal and plant cells, we also conducted evaluations on other samples to analyze the universality of the algorithm, as shown in Figure 10. Figures (i) and (iii) are wide-field images, and Figures (ii) and (iv) are DM-SIM-LLIE images. Figures (iii) and (iv) are magnified wide-field and DM-SIM-LLIE images of the same sample area, respectively, selected by the red frame. Firstly, we tested the performance of DM-SIM-LLIE on fluorescently labelled actin. Secondly, we selected a Pap smear sample. A Pap smear is a sample of exfoliated cervical cells. Thirdly, we selected a pathological tissue section sample. The experimental results for these three samples are shown in Figure 10a–c. The DM-SIM-LLIE method produced final super-resolution images with improved contrast.

## 5. Conclusions

This study focuses on three key challenges associated with structured illumination microscopy (SIM): poor performance when imaging weak light, low reconstruction efficiency, and insufficient axial resolution. The proposed spatial super-resolution algorithm, DM-SIM-LLIE, incorporates the following innovations:

Firstly, it incorporates an adaptive weak light enhancement module. Based on machine learning algorithms, this module dynamically optimizes pixel-level brightness and contrast, overcoming the dependence of traditional SIM systems on lighting conditions. It enables stable, high-quality, low-light imaging and the enhancement of various samples.

Secondly, the algorithm employs a temporal super-resolution reconstruction method. This algorithm uses spatiotemporal image correlations to enable rapid reconstruction in π/2 phase-shift SIM systems. Compared to traditional frequency-domain reconstruction methods, experimental data show that this algorithm achieves a 5.49-fold increase in reconstruction speed and nearly doubles lateral resolution, significantly enhancing real-time imaging efficiency.

Thirdly, the axial resolution has also been significantly improved. The DM-SIM-LLIE algorithm enhances the axial resolution of the SIM system on experimental samples from 580 ± 5 nm to 305 ± 5 nm. This performance is comparable to that of light-sheet microscopy, significantly expanding the range of applications for 3D imaging.

DM-SIM-LLIE demonstrates outstanding performance when imaging both mammalian and plant cells. It successfully resolves the balance between resolution, speed, and phototoxicity in biological imaging, offering new solutions for techniques such as live imaging, clinical pathology diagnosis, and 3D volume reconstruction. The algorithm has broad application prospects and research value.

## Figures and Tables

**Table 1:** Evaluation results of different methods in Figure 6.

Dataset	Metrics	SCI	EnGAN	ZeroDCE++	Kind++	RetinexNet	UnretNet	RetMamba	RUAS	Ours
CCPS	SSIM↑	0.856	0.159	0.509	0.677	0.061	0.582	0.193	0.878	0.893
	PSNR↑	19.941	19.477	14.958	16.263	16.714	15.586	16.440	20.255	20.490
	CII↑	2.888	2.731	3.294	4.163	2.027	4.337	3.831	2.745	2.702
	RMSE↓	25.673	27.081	45.568	39.206	37.225	42.386	38.415	24.763	24.102
MTs	SSIM↑	0.347	0.361	0.384	0.453	0.263	0.309	0.569	0.474	0.699
	PSNR↑	10.466	10.124	10.822	11.256	7.279	7.275	15.076	12.247	17.130
	CII↑	2.809	2.393	1.868	2.003	1.450	2.436	2.125	3.086	2.101
	RMSE↓	76.428	79.491	73.354	86.367	110.301	110.347	44.947	62.261	35.483
F-actions	SSIM↑	0.427	0.271	0.256	0.256	0.1799	0.295	0.274	0.576	0.782
	PSNR↑	11.126	10.363	10.441	11.472	8.603	7.549	8.582	12.926	18.451
	CII↑	3.942	3.367	3.769	3.769	3.518	4.211	4.904	3.311	2.194
	RMSE↓	70.831	77.340	76.640	76.369	94.708	106.917	94.928	57.578	30.478

**Table 2:** Running time of the tested algorithms.

Algorithm	Time/s
FDR-SIM FDR-SIM-LLIE	3.90 random
DM-SIM DM-SIM-LLIE	0.71 2.23

## Data Availability

Data is contained within the article: the original contributions presented in this study are included in the article. Further inquiries can be directed to the corresponding author.
